# Outpatient Antibiotic Dispensing for the Population with Government Health Insurance in Syria in 2018–2019

**DOI:** 10.3390/antibiotics9090570

**Published:** 2020-09-02

**Authors:** Saleh Aljadeeah, Veronika J. Wirtz, Eckhard Nagel

**Affiliations:** 1 Institute of Medical Management and Health Sciences, University of Bayreuth, Prieserstr. 2, 95440 Bayreuth, Germany; eckhard.nagel@uni-bayreuth.de; 2 Department of Global Health, Boston University School of Public Health, Boston, MA 02118, USA; vwirtz@bu.edu

**Keywords:** antibiotics, outpatient, dispensing data, Syria, demography

## Abstract

Little is known about antibiotic uses at the population level in Syria. The aim of our study is to present outpatient antibiotic dispensing (OAD) patterns and rates for patients with health insurance in the parts of Syria that are controlled by the Syrian government using different indicators. Outpatient data on all dispensed antibiotics for 81,314 adults with health insurance were obtained and stratified according to age, sex, governorate and annual season. OAD was mainly expressed as the number of defined daily doses (DDDs) per 1000 people per day (DID). OAD patterns were assessed according to the anatomical therapeutic classification (ATC) and the Access, Watch and Reserve (AWaRe) classification. OAD was 20.13 DID. Amoxicillin/clavulanic acid and clarithromycin were the most dispensed antibiotics (5.76 and 4.4 DID, respectively). Overall, a predominant consumption of broad-spectrum antibiotics was noted. The Watch group of the AWaRe classification had the biggest percentage of OAD (13.26 DID), followed by the Access and the Reserve groups (6.55 and 0.17 DID, respectively). There was a significant difference in OAD between the sex and age groups. The seasonal and regional variations in OAD were also significant. Broad-spectrum antibiotics dispensing was high compared to other studies from different countries. These results are concerning, as they can contribute to antibiotic resistance.

## 1. Introduction

Antibiotics play an essential role in controlling infectious diseases [1]. However, the use, including misuse and overuse, of antibiotics is one of the main factors contributing to the development of antibiotic resistance. This has reduced the treatment options for infectious diseases, jeopardizing global public health [2]. 

In Europe and North America, outpatient antibiotic dispensing (OAD) is often prescription-only [3] but can be accessed without prescriptions in many countries of the global South [4,5]. In 1988, Syria’s Ministry of Health legislated to determine which drugs could be sold over-the-counter. Antibiotics were not included [6]. However, a lack of enforcement of the laws and guidelines and community pharmacists’ financial interests have led to the common practice of dispensing antibiotics over-the-counter in Syria [7]. Another challenge related to the accessibility of antibiotics in Syria is linked to the ongoing conflict situation. This conflict has increased the prevalence of penetrating traumatic injuries with contaminated open wounds and fractures. Antibiotics are required to treat these injuries, usually for prolonged periods [8]. The conflict has also caused the epidemic spread of infections through vulnerable populations [9].

Health indicators have improved considerably in Syria, and the pharmaceutical sector developed successfully during the three decades before the conflict [10,11,12]. Since 2011, millions of civilians have borne the brunt of this conflict, with nearly one in three of the population (6.2 million out of 21 million) forced to leave their homes between 2011 and 2018. Two-thirds of the population in Syria live below the poverty line. The continuing conflict and internal displacement have had severe consequences for Syria’s health system [12]. Less than half of public health care facilities were fully functioning in 2018. Tens of thousands of injured Syrians have died or sustained lifelong disabilities, having been unable to access medical assistance [13].

Healthcare in Syria is financed by public funds and private insurance. Out-of-pocket expenditures on outpatient consultations and medicines is common [14]. No national health insurance is available to cover all inhabitants in Syria. However, individuals working in public organizations, some ministries and professional associations are provided with health insurance [15]. Private insurance companies have provided individual and group health plans since 2004 [16]. The total number of people with health insurance in Syria reached 841,852 by 2019; most of them (79%) are employed by the government [17].

The use of antibiotics has been intensively studied due to the public health consequences related to their widespread use [18]. Despite Syria’s high rates of antibiotic resistance and the need for antibiotic stewardship [8], insufficient data are available regarding the patterns of antibiotic consumption [19]. Few studies have examined nonprescription access to antibiotics in Syria, and those published were limited to certain settings, such as universities and or larger urban areas, e.g., Aleppo and Damascus [7,20,21]. None described the patterns or calculated the quantities of used or dispensed antibiotics.

This study aims to present OAD patterns and rates for patients with health insurance using different indicators. We performed additional analyses to explore potential associations between the antibiotic dispensing rates and demographic factors, such as age and sex, and assess any seasonal or regional variations.

## 2. Methods

### 2.1. Setting and Data Source 

This study is based on outpatient dispensing data from thirteen Syrian governates, excluding the Ar-Raqqa governorate, which is not under the Syrian government’s control. We used health insurance data covering 12 months from June 2018 to May 2019 to express annual OAD patterns and rates and to include only one influenza winter peak per the 12-month period [18]. Health insurance data included the drug name, number of dispensed units, pharmaceutical form and administration route of the drug, prescription number, date of dispensing, the name of the governorate where the antibiotic drug was dispensed and each patient’s age and sex.

### 2.2. Study Population

This study included the outpatient dispensing data of 81,314 beneficiaries. These beneficiaries were either employed by the Syrian government and covered by the health insurance scheme, members of professional associations or privately insured university students. Government employees belong usually to Syria’s middle-income class [22].

### 2.3. Data Analysis

The data in this study included all medicines dispensed for the study population. We included only medicines classified in the anatomical therapeutic chemical (ATC) classification as J01 (antibacterials for systemic use). We used the World Health Organization (WHO) ATC/DDD (defined daily dose) methodology to describe the antibiotic dispensing patterns (Version 2020). The defined daily dose (DDD) is “the assumed average maintenance dose per day for a drug used for its main indication in adults” [23]. DID stands for the number of defined daily doses per 1000 inhabitants per day. OADs were presented by pharmacological subgroup (ATC3) and chemical substance (ATC5). The relative dispensing of antibiotics was presented as a percentage of the total dispensing by route of administration (oral or parenteral) and AWaRe categories (Access, Watch and Reserve). The Access category includes 48 antibiotics that are associated with a lower potential for antimicrobial resistance and recommended as the first and second choices for treating infections. The Watch category includes 110 antibiotics with a higher potential for antimicrobial resistance. The Reserve category includes 22 antibiotics. The antibiotics of the Reserve group are considered as a “last resort” to treat infections with multidrug resistant bacteria. They should only be applied when all other alternatives have failed to treat an infection. Stewardship programs should focus on antibiotics of the Watch and Reserve groups [24]. Some dispensed antibiotic combinations were not listed in the WHO AWaRe classification. We classified this group of antibiotics as “Not classified”. We used the drug utilization 90% methodology to rank antibiotics by volume of DIDs and specify which antibiotics account for 90% of the total dispensing [25]. We presented the volume of the OAD using the following indicators: total number of packages, DDD and DDD per 1000 inhabitants per day (DID). We adjusted DID by patient characteristics (sex and age). To adjust the number of DID by governorate, we calculated the number of DDDs per 1000 dispensing events in each governorate per day (DDED), since one beneficiary could receive antibiotics in several governorates. We also adjusted the number of DIDs for season by calculating the number of DDDs per 1000 inhabitants per day (out of 91 days). 

### 2.4. Statistical Analysis

All statistical assessments were performed using IBM SPSS Statistics version 25 (SPSS Inc., Chicago, IL, USA) [26]. The significance of differences between the variables was assessed using nonparametric testing, as the dispensing data showed some evidence of a skewed distribution. A *p*-value of 0.05 was used as a cut-off for significance. We used the Mann–Whitney test to examine the significance of the differences between the medians of the OAD rates among females and males. We used the Kruskal–Wallis nonparametric ANOVA to examine the significance of the differences between the medians of the OAD rates in the different age groups, regions or seasons. 

### 2.5. Ethical Considerations

Each patient was given a unique identifier number, but individual patients could not be identified. We secured approval from the ethics commission of the University of Bayreuth, who stated that analysis and reporting of the dispensing data in this study did not require their approval.

## 3. Results

This study is based on the outpatient dispensed medicines for 81,314 beneficiaries covered by health insurance in Syria. All these beneficiaries are adults (18≤). Of these, 33,444 patients (41.13%) received at least one antibiotic as outpatients between 1 June 2018 and 31 May 2019. The patients’ mean age was 44.76 years: 22,383 (66.9%) were female, and 11,061 (33.1%) were male. The Supplementary Table S1 includes the number of patients in the different sex and age groups. The total number of the OAD packages reached 76,774. The highest number of antibiotic packages dispensed for a single patient was 22. The total number of OAD DDDs is 59,7518.81, and the total number of OAD DIDs was 20.13.

The most frequently dispensed antibiotic was amoxicillin/clavulanic acid (5.76 DID), followed by clarithromycin (4.4 DID). Nine antibiotics accounted for 90% of the total dispensed antibiotics (Table 1).

Considering the number of dispensed antibiotics packages, 85% of the OAD in this study were for oral administration. The remaining 15% were for parenteral administration. Considering the number of DIDs, 98.6% of OADs were for oral administration, and 1.4% were for parenteral administration. Amoxicillin/clavulanic acid was the most dispensed oral (28.96%), followed by clarithromycin (22.17%). Ceftriaxone was the most dispensed antibiotic for parenteral administration (34.27%), followed by ceftriaxone and sulbactam (31.32%). Cephalosporines were the most dispensed group of antibiotics (ATC4), followed by penicillins. Table 2 lists the OADs divided by the pharmacological groups

### 3.1. Variation of Dispensed Antibiotics According to Patients’ Demographic Characteristics

A statistically significant difference was observed in OAD rates between female and male patients (*p* = 0.003). The adjusted DID rates of OADs were higher among female patients (21.58 DID) than male patients (17.76 DID). 

A statistically significant difference was observed in OAD rates between the different age groups (*p* < 0.001). Adjusted OAD rates were the highest among the patients aged 30–39 (25.07 DID) and the lowest among the patients aged 60–69 (14.37 DID). 

The antibiotics in the Watch category of the AWaRe classification had the highest percentage of OAD in this study (65.86%), followed by Access antibiotics (32.54%). Antibiotics in the Reserve category had a smaller percentage (0.83%). The remaining antibiotics (0.76%) were not classified (Table 3).

### 3.2. Seasonal Variation

**Figure 1:**
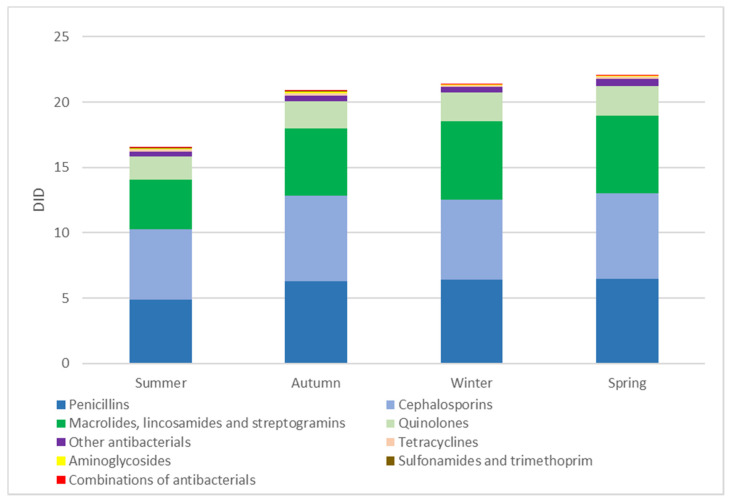
Total outpatient antibiotic dispensing rates (DID) among adults with health insurance in Syria by pharmacological group (ATC4) and season. DID: the number of defined daily doses (DDDs) per 1000 people per day.

A significant difference was observed in OAD rates between the different seasons (*p* < 0.001). OAD rates were highest in the spring (22.05 DID), followed by the winter (21.36 DID) and autumn (20.84 DID). The antibiotic dispensing rates were lowest in the summer (16.49 DID) (Figure 1).

### 3.3. Regional Variation

**Figure 2:**
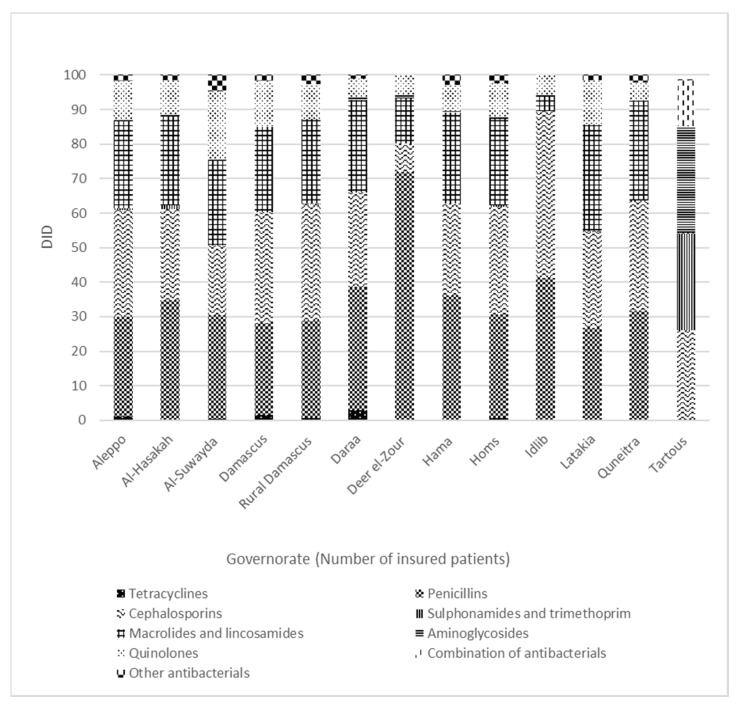
Proportional outpatient antibiotic dispensing (DID%) among adults with health insurance in Syria by pharmacological group (ATC4) and governorate.

**Figure 3:**
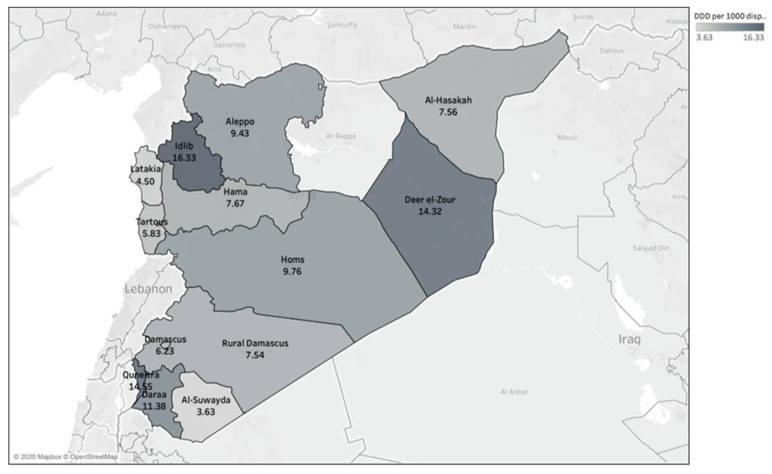
Adjusted outpatient antibiotic dispensing rates (DDD/1000 dispensing events/day) among adults with health insurance in Syria by governorate.

During the study period, a significant difference (*p* < 0.001) in the total OAD was observed between Syria’s different governorates that are under control of the Syrian government (Figure 2). According to the number of DDEDs in each governorate, Idlib (16.33 DDED), Quneitra (14.55 DDED) and Deer el-Zour (14.32 DDED) had the highest OAD rates, while Tartous (5.83 DDED), Lattakia (4.5 DDED) and Al-Suwayda (3.63 DDED) had the lowest OAD rates (Figure 3).

## 4. Discussion

To our knowledge, this is the first study to have used different indicators to describe the patterns and present the quantities of dispensed antibiotics using health insurance data with a large sample (81,314 beneficiaries) over a period of 12 months. 

Our results show that the total OAD rate reached 20.13 DID. Borg et al. have reported high levels of antibiotic consumption in hospitals in the southern and eastern Mediterranean regions [27] Many studies have reported over-prescriptions of antibiotics in middle- and low-income countries [28,29,30]. The WHO report on the Surveillance of Antibiotic Consumption showed that the total antibiotic consumption rates in Turkey and in Iran are among the three-highest antibiotic consumption rates of the 65 countries included in the study [31]. A comparison of the antibiotic dispensing rates in our study with those of such a report is not appropriate, because this report was not limited to outpatient dispensing data for adults, as in our study [29]. Studies reporting antibiotic dispensing in outpatient settings from low- and middle-income countries are scarce, making it difficult to compare our data with those of studies that used the same settings. Comparing the OAD rates in our study with the OADs in European countries, the rates in our study are higher than the average OAD rates in these countries in the years 2017/2018 (18.4 DID) [32]. We should mention that the OAD data from the European countries included children’s OAD data, which also limited the direct comparison of these data to those in our study. 

The WHO report showed that penicillins are the most dispensed antibiotics in 62 of the 65 countries included in the study [31], while cephalosporines were the most dispensed antibiotics in our study. This can be explained by these factors: (1) the high prevalence of antimicrobial resistance against penicillin in Syria [33], and (2) this study included only adults, while the WHO report included children. Amoxicillin consumption rates are usually high among children [34].

In this study, amoxicillin/clavulanic acid was the most frequently dispensed antibiotic (28.62%), followed by clarithromycin (21.87%) and cefixime (13.05%). The dispensing rates of these three broad-spectrum antibiotics account for over 50% of the total OAD in this study. This may again be linked to an increased resistance to narrow-spectrum antibiotics. Šahman-Zaimović et al. found that, in cases of high resistance to amoxicillin, amoxicillin/clavulanic acid is recommended [35]. The broad-spectrum antibiotic dispensing rates in this study are high. Further research is required, studies to determine whether the prescribing pattern aligns with the antimicrobial prescribing guidelines.

A statistically significant difference was observed in OAD rates between female and male patients. OAD was higher among female patients compared to male patients. Many studies from different parts of the world showed higher consumption or prescription rates among females than males [36,37,38,39]. 

The variation in OAD rates between the different age groups was statistically significant. A difference was observed in the patterns of OAD among the different age groups. The age variations in the patterns of OAD could be explained by the different infection types across the age groups. Several studies have shown variations in these patterns and a high prevalence of antibiotic use among elderly patients [37,40,41]. The adjusted OAD DID rates for age were lower among the patients aged over 60 years compared to younger patients. The reason for this result is uncertain, but it might be related to the conflict situation where younger people are most likely to be involved in the conflict and are more exposed to conflict-related injuries.

Considering the number of dispensed antibiotic packages, 15% of the OADs in this study were for parenteral administration, whereas according to the DID of the dispensed antibiotics, 1.4% were for parenteral administration. The variation in the proportion may be explained by the fact that a package of oral antibiotics has, on average, 9.02 DDDs, while parenteral antibiotic packages have, on average, 0.72 DDDs. Some physicians and patients still believe that parenteral antibiotics are more effective than oral ones [42,43]. Ceftriaxone was the most dispensed parenteral antibiotic (34.27%), followed by ceftriaxone and sulbactam (31.32%). Other studies have shown that ceftriaxone is the most common parenteral antibiotic in outpatient settings [44,45]. Ceftriaxone has a long half-life and requires one dose per day. As such, physicians and patients prefer ceftriaxone in outpatient settings [46]. The high proportion of broad-spectrum parenteral antibiotics in this study might, however, lead to more antibacterial resistance. The WHO does not recommend the use of the fixed-dose ceftriaxone and sulbactam combination on the grounds that it is not evidence-based nor recommended in high-quality international guidelines [24]. This presents evidence of the misuse of antibiotics in Syria.

The OAD of Watch antibiotics for the patients in this study was the highest (65.86% of total DID), followed by Access antibiotics (32.54%). The dispensing of Reserve antibiotics was the lowest (0.83%). The remaining antibiotics (0.76%) were not classified. Comparing these results to those from the WHO reporting the proportional consumption of antibiotics by AWaRe categorizations in three countries of the Eastern Mediterranean Region, 2015 (Iran, Jordan and Sudan), we notice that a smaller share of antibiotic consumption belongs to the Access group (32.54% in this study), while >53.8% of the antibiotics were in the Access group in Iran and 65.3% Sudan, and the share of Access was lower in Jordan (30.5%), with a larger share to the Watch group (65.86% vs. 45.0% in Iran, 17.1% in Sudan and 59.0% in Jordan), as well as to the Reserve group (0.83% vs. 0.1% in Jordan, while this was not reported in Iran or Sudan). The data in that study, unlike our study, were not limited to OAD data but included sales data and inpatient data [31]. This may show different proportions across the three groups. This result places Syria in one of worst positions in terms of the quality measures of the AWaRe classification system.

The dispensing data included seven fixed-dose antibiotic combinations. These accounted for 29.64% of the total OAD. Three of these fixed-dose combinations were listed in the Access group, and the remaining four combinations were not included in the AWaRe classification system (Table 4). These four combinations accounted for 0.82% of the total OAD. The WHO does not recommend the use of these combinations in clinical practice, as the use of these four fixed-dose combinations of multiple broad-spectrum antibiotics is not evidence-based nor recommended in high-quality international guidelines [24]. 

We noticed in the dispensing data that some beneficiaries have dispensed antibiotics in more than one governorate. Adjusting the OAD rates according to governorates by calculating the number of DDDS per 1000 inhabitants (of each governorate) per day can be misleading. Therefore, we decided to adjust the OAD rates according to governorates by calculating the number of DDDs per 1000 OAD events per day. We do not have a certain explanation for the regional variations in the adjusted OAD rates and patterns. The adjusted OAD rates were the highest in Idlib, Quneitra and Deer el-Zour. Major parts of Idlib and Deer el-Zour were out of the Syrian government’s control and were affected by the armed conflict during the study period. While the major parts of the three governorates with the lowest adjusted OAD rates (Al-Suwayda, Lattakia and Tartous) were under the Syrian government’s control and less affected by the conflict [47]. The variations of the adjusted OAD rates might be related to the conflict situation in Syria.

### Limitations and Strengths

This study included OAD for insured people in parts of Syria that are controlled by the Syrian government. The number of insured people in governorates such as Homs and Hamah was significantly higher than the number of insured people in the governorates of Idlib and Deer el-Zour, because large parts of these governorates are beyond the Syrian government’s control. The number of people with health insurance in each governorate included in our study does not correspond to the actual population numbers and represents a limitation in terms of our ability to extrapolate our findings to the entire population. The results of this study could be possibly generalized for adults in parts of Syria that are controlled by the Syrian government and who belong to the middle-income class. The regional variations of dispensed antibiotics in this study can be related to many factors—for example, the uneven distribution of health and medical services across geographical regions [11] and the conflict situation that rendered services damaged or unavailable [48]. The conflict situation in Syria posed a challenge to mapping the parts of Syria that were under the control of the Syrian government during the study period. Further studies are required to cover the areas of Syria that are beyond the control of the Syrian government. The data did not include antituberculosis agents. The reported prevalence of tuberculosis in Syria is low (19 per 100,000 inhabitants in 2017) [49].

The data did not include the OAD that were prescribed to treat certain diseases that were excluded from health insurance coverage in Syria. The treatment for sexually transmitted diseases (STDs) or dental conditions were not covered by health insurance [50]. This raises the question of the appropriateness of policies regulating access to medicines, particularly antibiotics. We cannot claim that our study’s results represent either the OAD or the total consumption or prescription of antibiotics across the entire population. To represent Syria’s total antibiotic consumption, further data that includes pharmacy sales data, inpatient data, antibiotic consumption data for children and people without health insurance in regions under and outside the Syrian government’s control are required.

The limited possibilities of aggregating or accessing data on antibiotics usage in Syria is a major challenge in conducting a study that aims to be representative of the entire population. This study represents a pioneering step toward the complete picture of antibiotic dispensing in Syria. It may function as a starting point for future studies that will yield a greater understanding of the prescriptions and uses of antibiotics in Syria. A major strength of this study is its use of different indicators that are often used to describe the patterns of antibiotics usage, allowing comparisons of the results of this study with those of studies describing OAD. This study describes antibiotic dispensing patterns for a part of the population in a region that is usually underrepresented in studies on antibiotics use. 

## 5. Conclusions

This study provides the first estimates of OAD rates and patterns of a population with health insurance represented by 81,314 adults across areas controlled by the Syrian government. We observed high rates in the OAD of broad-spectrum antibiotics and high percentages of the Watch group of the AWaRE classification. These results are concerning, as the consumption of the Watch group does significantly increase the resistance against antibiotics. Further efforts are needed to investigate the quality of antibiotic use in different settings and among different groups of Syria’s population. Our study provides critical findings that may help improve health insurance policies, antibiotics prescription guidelines and stewardship programs. We therefore encourage further research that would report antibiotic dispensing or consumption patterns and rates in different settings and population groups across Syria.

## Figures and Tables

**Table 1:** Outpatient antibiotic dispensing rates that accounted for 90% of total dispensed antibiotics among adults with health insurance in Syria expressed in the number of defined daily doses (DDDs) per 1000 people per day (DID). AWaRE: Access, Watch and Reserve.

Number	Antibiotic	AWaRe	DID	Proportion of Total DID
1	Amoxicillin/Clavulanic acid	Access	5.76	28.62%
2	Clarithromycin	Watch	4.4	21.87%
3	Cefixime	Watch	2.66	13.05%
4	Cefuroxime	Watch	1.85	9.17%
5	Levofloxacin	Watch	1.09	5.4%
6	Azithromycin	Watch	0.72	3.59%
7	Cefdinir	Watch	0.63	3.15%
8	Cefprozil	Watch	0.55	2.71%
9	Ciprofloxacin	Watch	0.51	2.55%
Drug Utilization 90% (DU90%) 1–9			18.14	90.11%
Others 10–53			1.99	9.89%
Total			20.13	100%

**Table 2:** Total outpatient antibiotic dispensing rates among adults with health insurance in Syria according to the pharmacological group (ATC4) and expressed in DID.

Pharmacological Group	DID	Proportion of Total DID
Cephalosporins (J01D)	6.14	30.49%
Penicillins (J01C)	6.01	29.87%
Macrolides, lincosamides and streptogramins (J01F)	5.19	25.78%
Quinolones (J01M)	2.08	10.31%
Other antibacterials (e.g., linezolid, metronidazole and nitrofurantoin) (J01X)	0.46	2.29%
Tetracyclines (J01A)	0.14	0.6%
Aminoglycosides (J01G)	0.06	0.29%
Sulfonamides and trimethoprim (J01E)	0.04	0.21%
Combinations of antibacterials (J01R)	0.01	0.06%
Total	20.13	100%

**Table 3:** Total outpatient antibiotic dispensing rates among adults with health insurance in Syria according to the AWaRe classification and expressed in DID.

AWaRe	DID	Proportion of Total DID
Access	6.55	32.54%
Watch	13.26	65.86%
Reserve	0.17	0.83%
Nonclassified	0.15	0.77%
Total	20.13	100%

**Table 4:** Dispensed outpatient antibiotic combination rates among adults with health insurance in Syria classified by AWaRe categories and expressed in DID.

Combination of Antibiotics	AWaRe	DID	Proportion of the Total DID of Antibiotic Combinations
Amoxicillin + clavulanic Acid	Access	5.76	96.51%
Ceftriaxone + Sulbactam	Not Recommended	0.09	1.47%
Amoxicillin + Flucloxacillin	Not Recommended	0.06	1.08%
Sulfamethoxazole + Trimethoprim	Access	0.04	0.7%
Spiramycin + Metronidazole	Not Recommended	0.01	0.2%
Ampicillin + Cloxacillin	Not Recommended	0.002	0.04%
Ampicillin + Sulbactam	Access	0.0001	0.002%
Total		5.96	100%

## Supplementary Materials

The following are available online at https://www.mdpi.com/2079-6382/9/9/570/s1, Table S1: The number of patients in this study according to the sex and age groups.
