# Supplementary material for: Outpatient Antibiotic Dispensing for the Population with Government Health Insurance in Syria in 2018–2019
Source: Antibiotics (Basel). 2020 Sep 2;9(9):570. doi: 10.3390/antibiotics9090570 (PMC7559287; doi:10.3390/antibiotics9090570)
Supplement: Supplementary file 1 [file antibiotics-09-00570-s001.pdf]

Supplementary Materials:

Table S1: The number of patients in this study according to sex and age groups.

**Age group (years)**	**Female**	**Male**	**Total**
18-29	3,036	500	3,536
30-39	6,994	2,187	9,181
40-49	5,620	3,470	9,090
50-59	4,997	3,103	8,100
60-69	1,533	1,234	2,767
70-79	188	519	707
80<=	15	48	63
Total	22,383	11,061	33,444
